# Participatory epidemiology of endemic diseases in West African cattle – Ethnoveterinary and bioveterinary knowledge in Fulani disease control

**DOI:** 10.1016/j.onehlt.2018.03.001

**Published:** 2018-03-27

**Authors:** Ayodele O. Majekodunmi, Charles Dongkum, Christopher Idehen, Dachung Tok Langs, Susan C. Welburn

**Affiliations:** a Division of Infection and Pathway Medicine, School of Biomedical Sciences, Edinburgh Medical School, College of Medicine and Veterinary Medicine, The University of Edinburgh, 1 George Square, Edinburgh EH8 9JZ, UK; b Livestock and Poultry Research Centre, University of Ghana, P.O. Box LG 25, Legon, Accra, Ghana; c Trypanosomiasis Department, Nigerian Institute for Trypanosomiasis Research, P. M. B. 1303, Vom, Plateau State, Nigeria; d Veterinary Clinic, National Veterinary Research Institute, P. M. B. 01, Vom, Plateau State, Nigeria

**Keywords:** Fulani, Pastoralist, Cattle, Participatory epidemiology, Participatory diagnosis, Ethnoveterinary knowledge

## Abstract

Fulani pastoralists in Nigeria lack adequate access to good quality veterinary services and often resort to treating their animals themselves. There are several negative aspects to this, including poor treatment outcomes, misuse of veterinary drugs and subsequent resistance, and further barriers to good relations between pastoralists and veterinary services. A participatory epidemiology survey was undertaken in Fulani communities, to examine their ability to diagnose and treat bovine diseases. Qualitative participatory epidemiology techniques including semi-structured interviews, ranking and participant and non-participant observations were used for data collection. Quantitative analysis to match Fulani disease descriptions to veterinary diseases was done by hierarchical clustering and multi-dimensional scaling. A concurrent parasitological survey for soil-transmitted parasites, trypanosomiasis and tick-borne diseases was undertaken to validate results.

Fulani pastoralists displayed high levels of ethnoveterinary knowledge and good clinical diagnostic abilities. Diseases considered important by pastoralists included: *hanta* (CBPP); *sammore* (trypanosomiasis); *boro* (foot and mouth disease), *gortowel (liver fluke)*, *dauda* (parasitic gastro-enteritis with bloody diarrhoea) and *susa* (parasitic gastro-enteritis). The parasitology survey supported the participatory epidemiology results but also showed a high prevalence of tick-borne diseases that were not mentioned by pastoralists in this study. The use of “*hanta*” to describe CBPP is important as the accepted translation is liver-fluke (*hanta* is the Hausa word for liver). *Gortowel* and *dauda*, two previously undescribed Fulfulde disease names have now been matched to liver fluke and PGE with bloody diarrhoea. Fulani showed low levels of bovine veterinary knowledge with mostly incorrect veterinary drugs chosen for treatment. Levels of ethno- and bio-veterinary knowledge and their application within pastoralist livestock healthcare practices are discussed.

## 1 Introduction

The livelihoods of Fulani pastoralists in Nigeria are heavily dependent on the health and productivity of their livestock. The livestock sector is important to the national economy, contributing to both financial and nutritional needs of the country through meat, milk and hides (6–8% of GDP) [1]. Veterinary services in Nigeria fail to meet the animal health needs of Fulani who have poor access to veterinary products and services [[2], [3], [4], [5], [6], [7], [8], [9]]. Disease surveillance, extension practice and veterinary service delivery are affected by a range of structural issues in Nigeria ranging, from failings in logistics to a lack of engagement with end users. The high cost of delivering veterinary services to rural and mobile communities is prohibitively expensive. Most veterinarians have high career expectations and are based in towns and cities offering fixed-point veterinary services Cultural and professional biases also impact on service provision to pastoralist communities as veterinarians with poor understanding of ethnoveterinary knowledge (EVK) and pastoral production systems are unable to engage effectively with pastoralists to deliver animal healthcare. Pastoralist communities have had bad experiences with fake or substandard drugs in the marketplace and poor-quality animal health services resulting in low trust and limited demand for services from outside of the community [2,4,[7], [8], [9]]. For pastoral systems in Nigeria, the biggest issues are with engagement because even if unlimited resources were available, they would not yield good results unless these problems were dealt with.

Endemic diseases of livestock are a major constraint to animal health, livestock production and rural economies. Control and surveillance has been progressively scaled back in many developing countries in favour of emerging, transboundary and zoonotic diseases [9]. While surveillance is a public sector responsibility, this is mostly done passively, especially in resource-poor pastoral settings where livelihoods depend heavily on livestock [8,10]. Endemic disease management is left to livestock owners and private sector service providers.

Participatory epidemiology (PE) emerged in the 1980s, offering a new method to rapidly survey for diseases and enable disease prioritization against a background of poor to non-existent veterinary services and disease surveillance [[11], [12], [13]]. Participatory epidemiology has also been successfully applied at the One Health interface for wildlife, biodiversity and natural resource management [[14], [15], [16]]. It is based on indigenous knowledge (IK), specifically ethnoveterinary knowledge (EVK) and the need to incorporate it with scientific knowledge (SK), specifically bioveterinary knowledge (BVK) for added benefits in disease surveillance, control and community based animal health (CBAH) systems [17,18]. This should result in a complementary, synergistic relationship between both knowledge systems which is acceptable to pastoralists, professionals and researchers.

Much of the literature focuses on the differences between the two knowledge systems, setting up a dichotomy in which IK is perceived as qualitative, subjective and contextual while SK is quantitative, objective and global [19,20]. However, scientists and researchers must take into account the wealth of evidence for the social and contextual dimensions of SK – it is just one of several available and competing knowledge systems and like all knowledge is socially constructed and situated in specific contexts [[21], [22], [23], [24], [25], [26]].

There are different approaches to working with these different knowledge systems. The “integration” approach focuses on “translating” IK into terms compatible with SK so that it can be integrated *into* SK. However, in this process, IK is distilled, compartmentalised and taken out of context, losing much of its value along the way. The “bridging” discourse recognizes these shortcomings and starting from a position of equality between knowledge systems, seeks to build bridges between the two epistemologies through a better understanding of how they differ. Focusing on their differences and similarities. The “dialogues” discourse is concerned with mutual exchange between the different knowledge spaces and focuses more methodologies and direct comparisons. However, this requires a good understanding of the underlying world view of each knowledge system [19,20,[27], [28], [29], [30]].

PE has expanded rapidly in Asia and Africa [12,31] especially within community based animal health (CBAH) systems in Eastern Africa [17,32,33]. Much of the work on PE and its use in CBAH systems has been done in East Africa with pastoral groups such as Maasai, Afar, Samburu, Turkana, Karamojong, etc. [[16], [17], [18],^32^,[34], [35], [36], [37], [38], [39]]. The Fulbe or Fulani are the largest pastoral group in Africa, numbering over 25 million, with ~40% of them living in Nigeria. Yet, relatively little has been written about participatory epidemiology with this group. In Nigeria, treatment of endemic livestock disease is mostly undertaken by Fulani themselves, drawing on both EVK and BVK [40,41]. This “pluralist” veterinary knowledge, which may be complementary and/or competitive, is framed by individual and socio-cultural factors that interact to shape health outcomes and knowledge transmission. Consensus and competence of livestock owners needs to be assessed in any study of pluralist veterinary knowledge and practices [37]. Most studies on EVK have focused on ethnobotany/ethnopharmacology rather than integrative animal health management which is the primary concern for pastoralists [[42], [43], [44], 37]. The few studies on pluralism in veterinary healthcare have identified high levels of EVK (including surgery, pharmacology and toxicology) amongst pastoralists across Africa, indicating a higher competence and consensus in EVK than in BVK amongst pastoralist [37,38,44].

There are clear gaps in our knowledge of current EVK methods used by Fulani pastoralists, how this interacts with BVK and how both knowledge systems influence Fulani ability to diagnose and treat endemic diseases in their livestock and their interactions with the veterinary services. This study has employed PE to try to answer these questions amongst Fulani in Nigeria. An epidemiological survey of endemic parasitic diseases of cattle was also conducted for confirmation/triangulation.

## 2 Materials and methods

### 2.1 Study area

This study was conducted in Bokkos Local Government Area (LGA) on the Jos Plateau, Nigeria. There over a million cattle in the area, ~70% managed by settled Fulani pastoralists who practice seasonal transhumance in both dry and wet seasons [45]. Village selection was purposive as a result of persistent insecurity and violence between members of different tribes and religions on the Jos Plateau since January 2010 [46,47]. Bokkos LGA was chosen as the study area for this project as it was relatively peaceful and secure. Despite the absence of ethnic/religious violence, armed robberies and cattle thefts affecting both indigenes and Fulani were common in Bokkos LGA.

### 2.2 Study design

The participatory epidemiology survey was carried out six villages (Bokkos, Daffo, Maiyanga, Mangar, Hurti, and Tambes) alongside an epidemiological survey on endemic disease control in cattle [48]. Within each study village, six household herds were selected for screening. Study site selection was purposive, based on security, previous prevalence of AAT [45], similar environmental conditions and husbandry practices. Household selection within villages was also purposive, based on willingness to participate and even geographical coverage of the village area. Enrolled animals were ear tagged and their identification data (i.e., ear tag number, breed, sex, coat colour, and age as given by owner at enrolment time) were recorded in individual files. Sampling began in April–May 2013, and was repeated at 3-month intervals thereafter until March 2013 to give 5 sampling periods.

Between April 2012 and March 2013, six herds of 80 animals each were selected in each of the study villages, a total of 480 animals per village and a total of 2880 animals across the study area. Enrolled animals were ear tagged and their identification data (i.e., ear tag number, breed, sex, coat colour, and age as given by owner at enrolment time) were recorded individually.

### 2.3 Participatory epidemiology methods

Longitudinal study design Between April 2012 and March 2013 data on endemic diseases of pastoral cattle was collected using participatory diagnosis and epidemiology methods. This included ranking, case histories, in-depth semi-structured interviews and key informant interviews. Interviews were conducted with herders in selected households and key informants amongst local vets and para-veterinarians. During the interviews, respondents were asked to list and rank the six most important diseases of local cattle and describe the clinical signs of these diseases. In addition, pastoralists were asked to list the number of cases, deaths and treatments used for each disease over the past 12 months. Interviews were conducted in Hausa.

### 2.4 Epidemiological survey

#### 2.4.1 Blood sample collection and DNA extraction

At each sampling point, 5 ml of blood was taken from the jugular vein of each animal. 1 ml of the collected blood was immediately dispensed into a Hemocue microcuvette to determine haemoglobin (Hb) concentration. 1 ml of the remaining collected blood was spotted onto an FTA matrix (Whatman Bioscience, Cambridge, UK) while still in the field and air-dried. All samples were placed in foil pouches with a silica desiccant for transport.

Five individual 3 mm discs were excised from each card for each individual animal sampled using a Harris Micropunch© (Whatman, UK). To avoid cross contamination between samples, five discs were punched from blank filter cards after each sample. The five 3 mm discs of blank filter paper were included as negative controls for the DNA extraction process. The FTA discs were washed twice for 15 min using 1 ml of Whatman FTA purification reagent to remove haemoglobin, discarding used reagent after each wash. FTA cards were then washed twice for 15-min in TE buffer (10 mM Tris, 0.1 mM EDTA, pH 8.0) to remove the FTA purification reagent and again the used buffer was discarded after each wash. FTA discs were dried for 30 min in an oven at 37 °C. Chelex suspension (100 μl of 5%) was added to the dry discs and discs were incubated at 90 °C for 30 min to elute DNA from the FTA discs. Eluted DNA was used to seed subsequent PCR reactions being found to be more sensitive than using a dried FTA disc as recommended by the manufacturers [[18], [19], [20]].

#### 2.4.2 Detection of tick-borne infections by PCR – RLB method

After preparation, each sample was subjected to PCR amplification according to the method of [48]. Briefly, three primer sets were used simultaneously to target the variable region in the 16S ribosomal RNA gene for *Ehrlichia*/*Anaplasma* and *Rickettsia* spp. detection, and in the 18S ribosomal RNA gene fragment for *Babesia*/*Theileria* detection. After amplification, the PCR-products obtained from each sample were hybridized on a blot on which *Anaplasma*, *Ehrlichia*, *Babesia* and *Theileria* species-specific oligonucleotide probes were covalently linked. After stringent washing to remove unbound PCR products, the hybridized PCR products were visualized using chemiluminescence.

#### 2.4.3 Detection of trypanosome infections by ITS PCR

PCR was carried out in 25 ml reaction mixtures containing 10× reaction buffer (670 mM Tris-HCl pH 8.8, 166 mM (NH4)2SO4, 4.5% Triton X-100, 2 mg/ml gelatin) (Fisher Biotech), 2 mM MgCl_2_, 200 lM of each of the four deoxynucleoside triphosphates (dNTPs), primers at 1 lM and 0.5 U of Taq DNA polymerase (Fisher Biotech) and 5 μl sample DNA. PCR cycles were: initial step at 94_C for 5 min, followed by 35 cycles of 94_C for 40 s, 58_C for 40 s, 72_C for 90 s, and final extension at 72_C for 5 min. DNA was amplified using a Dyad Peltier thermal cycler © (MJ Research Inc. USA). ITS1 CF: 5′ CCGGAAGTTCACCGATATTG 3′; ITS1 BR: 5′ TTGCTGCGTTCTTCAACGAA 3′ [49]. 15 μl of the PCR product was run on a1.5% agarose gel stained with GelRed© (Biotium, USA) alongside a 100 bp graduation marker at 100 V for 1 h on a Bio Rad Power Pac 300 machine. The gel was examined on a Gel Doc 2000© Bio-Rad using Quantity One© Bio-Rad software.

#### 2.4.4 Fecal sample collection and analysis

Fecal samples were collected from each animal per rectum at each sampling time and immediately transported to the field laboratory. Simple flotation, McMaster method and sedimentation tests were carried out on each sample. Approximately 5 g of fecal material were collected directly from the rectum of each study animal at each sampling time and immediately transported to the field laboratory station. Each sample was treated according to a protocol of simple flotation and McMaster method using saline solutions; and sedimentation to identify trematode eggs using tap water. Nematode eggs in the fecal samples were then identified to the genus level using standard parasitological criteria) [50,51].

### 2.5 Statistical analysis

Prevalence and mortality rates were calculated from the number of cases and deaths reported for each disease. Diseases were further ranked by “importance” – a composite value determined by triangulation of the rank assigned by respondents and frequency with which it was reported.

A comprehensive list of signs of illness used by Fulani and vets to identify sick animals was compiled for comparison of clinical diagnosis by both groups. Fulani disease descriptions were tested for agreement amongst pastoralists using Kendall’s W. They were then compared with standard veterinary descriptions of common endemic diseases to determine agreement between diagnosis and control methods of pastoralists and veterinarians in [35,52].

This analysis was done in SPSS software [53] Hierarchical clustering and Multidimensional scaling. Hierarchical clustering was conducted using the squared Euclidean distance as a measure of similarity between diseases/treatments and average linkage as the clustering method. Multidimensional scaling was conducted using the squared Euclidean distance as a measure of similarity between diseases/treatments with distances calculated from ordinal data. No disease similar to anthrax was described and it was added as a control in these analyses.

## 3 Results

### 3.1 Diagnosis

**Fig. 1:**
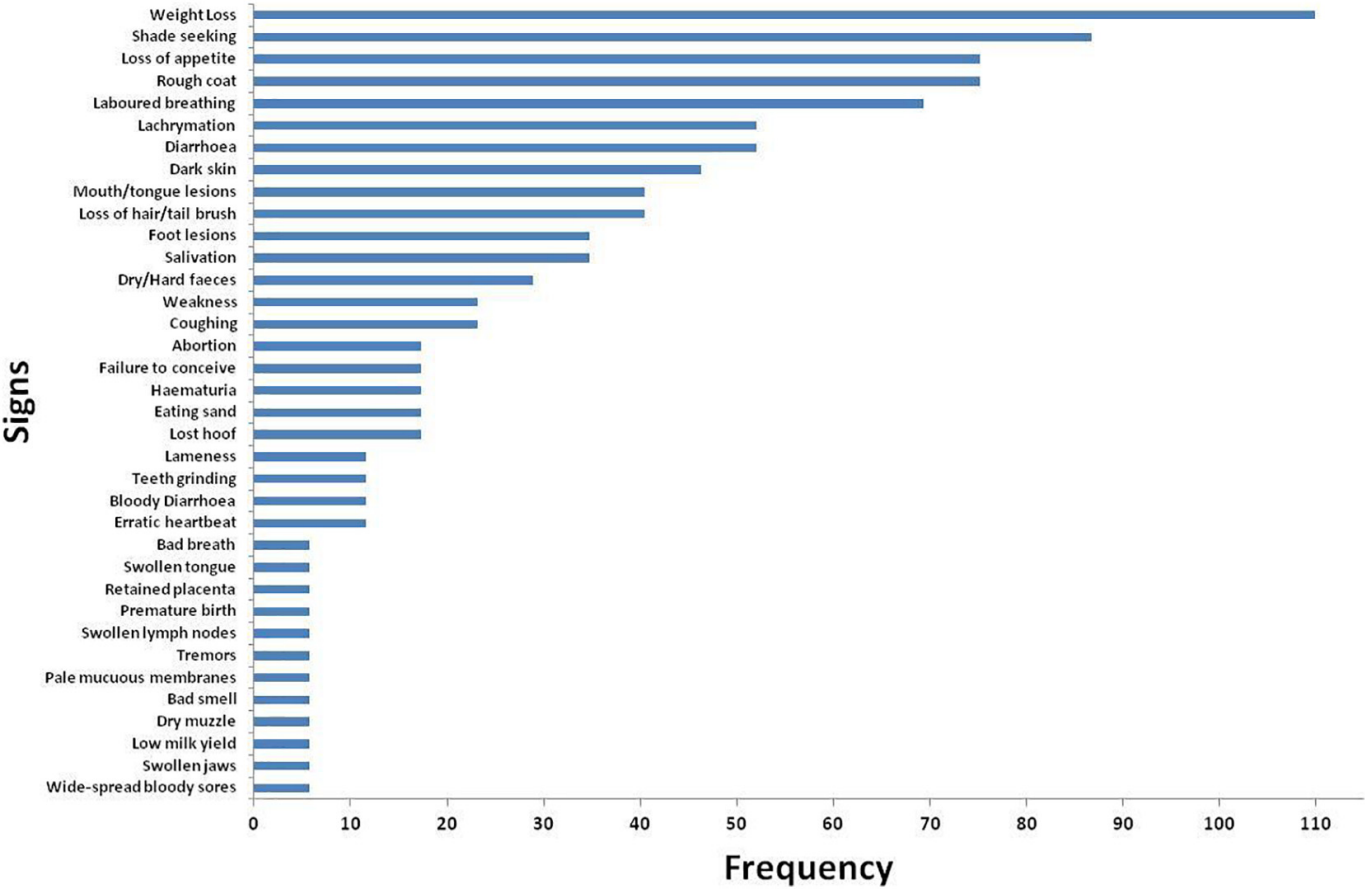
Signs of disease used in clinical diagnosis by Fulani.

Thirty-six distinct clinical signs of disease were reported by pastoralists in this study, the most common being weight loss, loss of appetite, shade seeking and rough or staring coats (see Fig. 1).

**Fig. 2:**
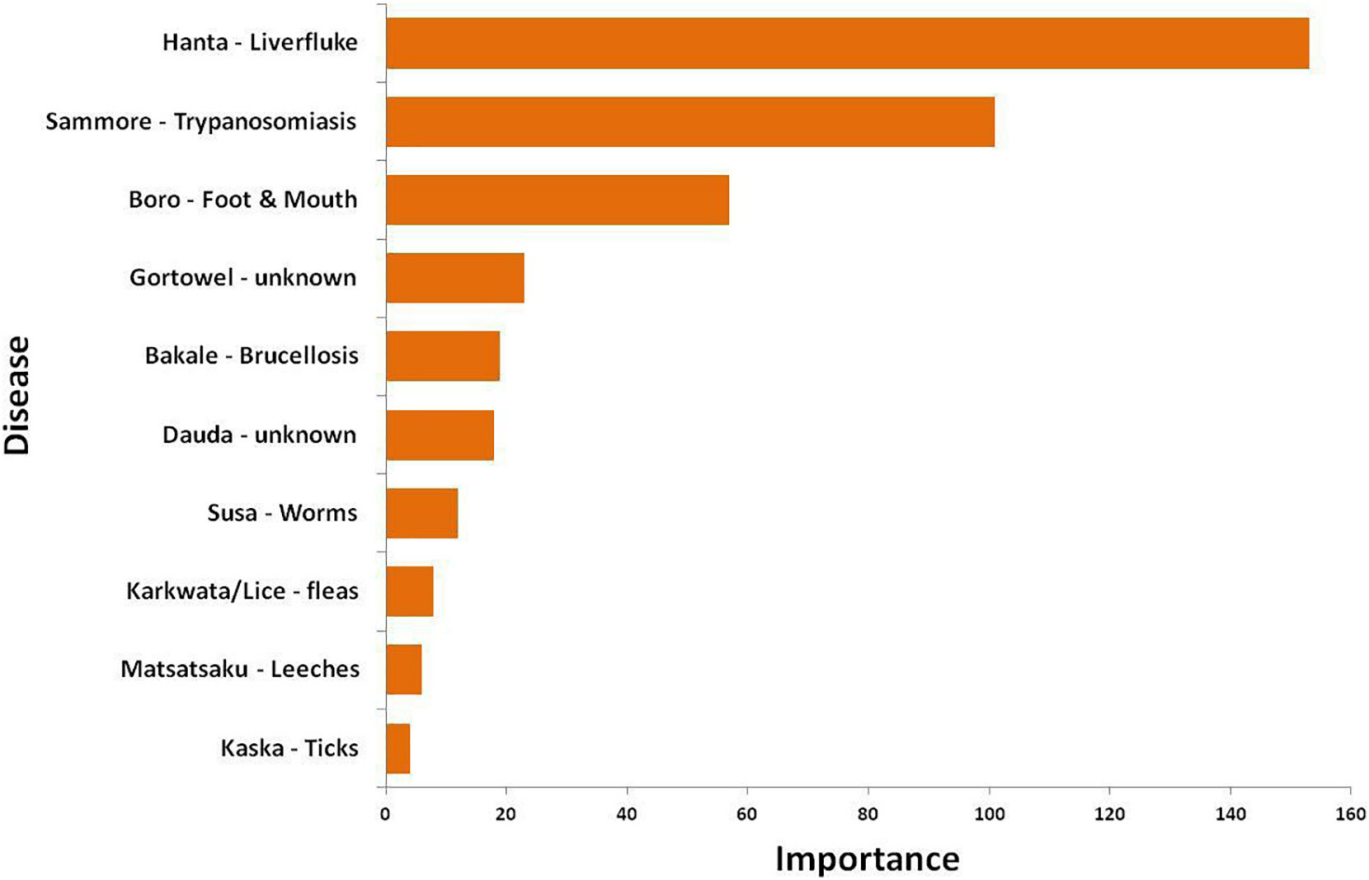
Diseases reported in Hausa with standard English translations.

According to the pastoralists interviewed in this study, the most important disease in their cattle was *hanta* (known as liverfluke) followed by *sammore* (trypanosomiasis), *boro* (foot and mouth disease), *dauda* (unknown)*, gortowel* (unknown)*, bakale*(brucellosis) *and* infestation by ectoparasites such as lice, fleas and ticks. All diseases were named in Hausa except for dauda and gortowel which are Fulfulde words (see Fig. 2). Of the 5835 cattle owned by the respondents in this study, 6.4% were reported by pastoralists to have *hanta*, 8.4% *sammore*, 37.9% *boro*, 3.8% *susa*, 0.4% *dauda*, 0.8% *gortowel* and 1.8% *bakale*.

**Fig. 3:**
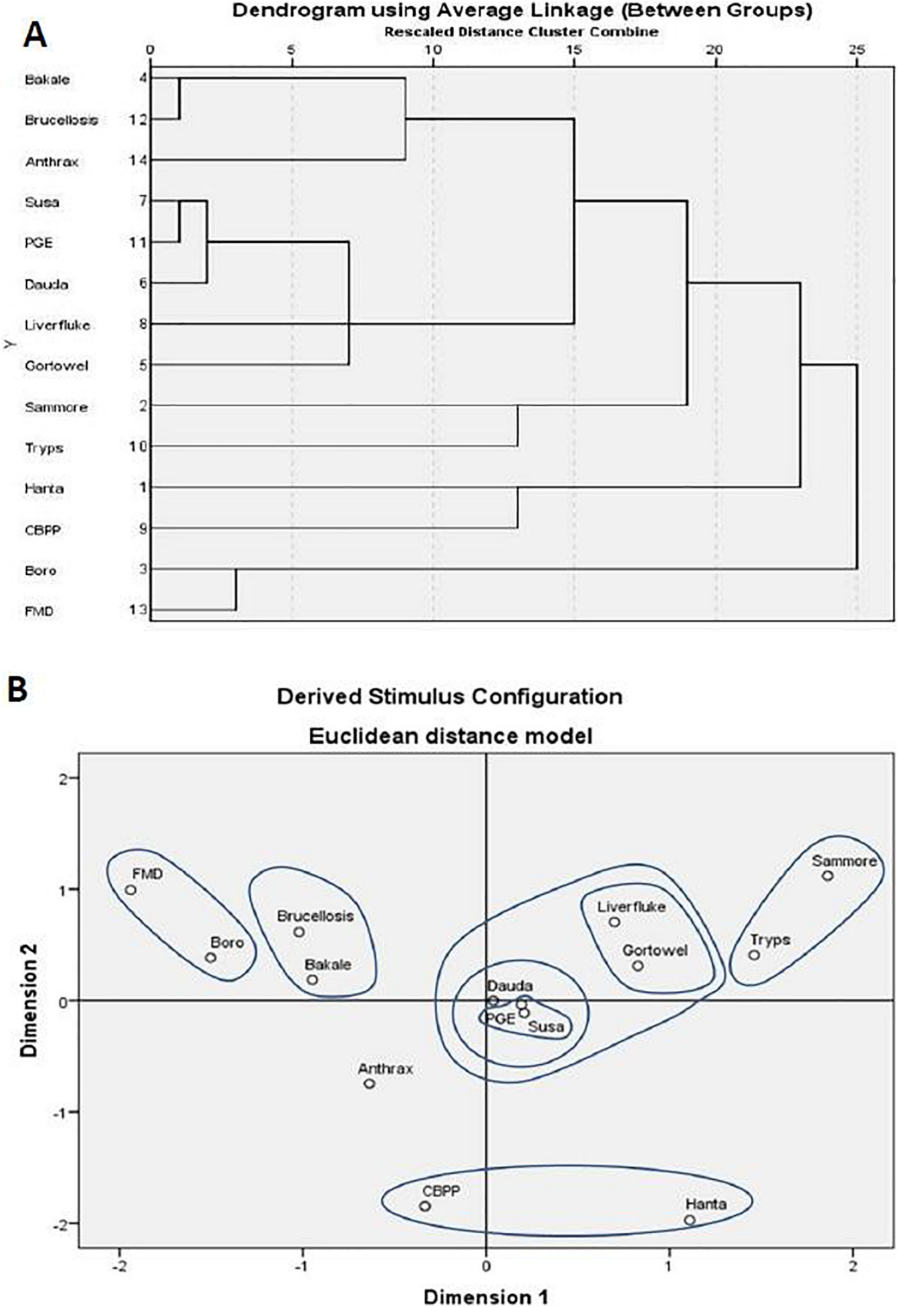
Comparison of herders' disease descriptions with standard veterinary descriptions using A: hierarchical cluster analysis and B: multidimensional scaling.

Table 1 shows disease descriptions and treatments. There was significant agreement for all disease descriptions (p < 0.05), however agreement levels were low amongst pastoralists with Kendall’s W scores below 0.3 for all diseases except Boro (moderate agreement, W = 0.385) and Bakale (strong agreement, W = 1). When compared with the standard veterinary descriptions of these diseases using hierarchical clustering and multidimensional scaling, most disease pairings were as expected considering standard translations of Hausa disease descriptors (Fig. 3); *Sammore* corresponding to trypanosomiasis; *boro* to foot and mouth disease (FMD); *bakale* to brucellosis and *susa* to parasitic gastroenteritis (PGE). However, *Hanta* paired with contagious bovine pleuropneumonia (CBPP - known as *huhu* in Hausa) rather than liver fluke. Previously unknown disease names *gortowel* and *dauda* (Fulfulde words for which the respondents had no Hausa equivalent) paired with liver fluke and PGE respectively. *Susa*, *dauda* and *gortowel* were closely linked, forming a super-cluster of gastro-intestinal helminths diseases. Anthrax, included as a control, did not match any reported disease.

**Table 1:** Disease descriptions and treatment profiles with paired veterinary diseases.

Disease	Signs reported by Fulani	Matching disease	Drugs by pairing
*Hanta*	Weight loss	**CBPP**	**CBPP**
	Shade seeking	Weight loss	Tylosine^a^
	Rough coat	Shade seeking	Long acting Oxytetracycline^a^
	Loss of appetite	Laboured breathing	CBPP Vaccine^a^
	Breathing difficulty	Salivation	Albendazole^b^
	Diarrhoea	Coughing	Levamisole^b^
	Lachrymation	Physical weakness	Closantel^b^
	Dry/Hard feces	Tremors	Levamisole/Oxyclozanide^b^
	Coughing		Nitroxyinil^b^
	Physical weakness		Diminazene
	Tremors		Isometamidium
	*W* = 0.179		Unnamed drugs/mixtures
			Herbs
*Sammore*	Weight loss	**Trypanosomiasis**	Diminazene^a^
	Rough coat	Rough coat	Isometamidium^a^
	Loss of appetite	Lachrymation	Albendazole
	Diarrhoea	Loss of hair/tail brush	Long acting Oxytetracycline
	Lachrymation	Weakness	Tylosine
	Loss of hair/tail brush	Abortion	Levamisole
	Swollen jaws	Pale mucous membranes	Closantel
	Pale mucous membranes	Swollen lymph nodes	Unnamed drugs/mixtures
	Swollen lymph nodes		
	Retained placenta		
	*W* *=* 0.235		
*Boro*	Weight loss	**FMD**	Dexamethasone^a^
	Loss of appetite	Loss of appetite	Procaine^a^
	Mouth and tongue blisters	Mouth/tongue lesions	Long acting Oxytetracycline
	Salivation	Salivation	Vitamins
	Foot ulcers	Foot lesions	Salt lick
	Lost hoof	Lost hoof	Unnamed drugs/mixtures
	Lameness	Lameness	
	*W* = 0.385	Low milk yield	
*Bakale*	Failure to conceive	**Brucellosis**	None^a^
	Abortion	Failure to conceive	Sulfadimidine
	*W* = 1.0	Abortion	Unnamed drugs/mixtures
		Retained placenta	
*Gortowel*	Weight loss	**Liver-fluke**	Diminazene
	Rough coat	Rough coat	Isometamidium
	Diarrhoea	Diarrhoea	Unnamed drugs/mixtures
	Lachrymation	Swollen jaws	
	Loss of hair/tail brush	Low milk yield	
	Low milk yield	Pale mucous membranes	
	*W* = 0.273		
*Dauda*	Rough coat	**PGE**	Long acting Oxytetracycline
	Diarrhoea	Weight Loss	Unnamed drugs/mixtures
	Bloody diarrhoea	Rough coat	Herbs
	*W* = 0.111	Loss of appetite	
		Diarrhoea	
		Bloody diarrhoea	
*Susa*	Weight loss	**PGE**	Albendazole^a^
	Rough coat	Weight Loss	Levamisole/Oxyclozanide^a^
	Diahorrea	Rough coat	
	*W* = 0.111	Loss of appetite	
		Diahorrea	
		Bloody diahorrea	

a Correct treatments.

b Correct treatments for liver-fluke.

Hierarchical clustering and multidimensional scaling showed high association between PGE, *susa* and *dauda*, as well as *gortowel* – liver fluke, brucellosis – *bakale* and FMD – *dauda*. There was less proximity between trypanosomiasis and *sammore* on hierarchical cluster analysis even though they grouped together in both dimensions in multi-dimensional scaling. The correlation of CBBP and *hanta* was weaker with both methods with early divergence in the cluster on HC and grouping along only 1 dimension on MDS.

### 3.2 Treatment

**Fig. 4:**
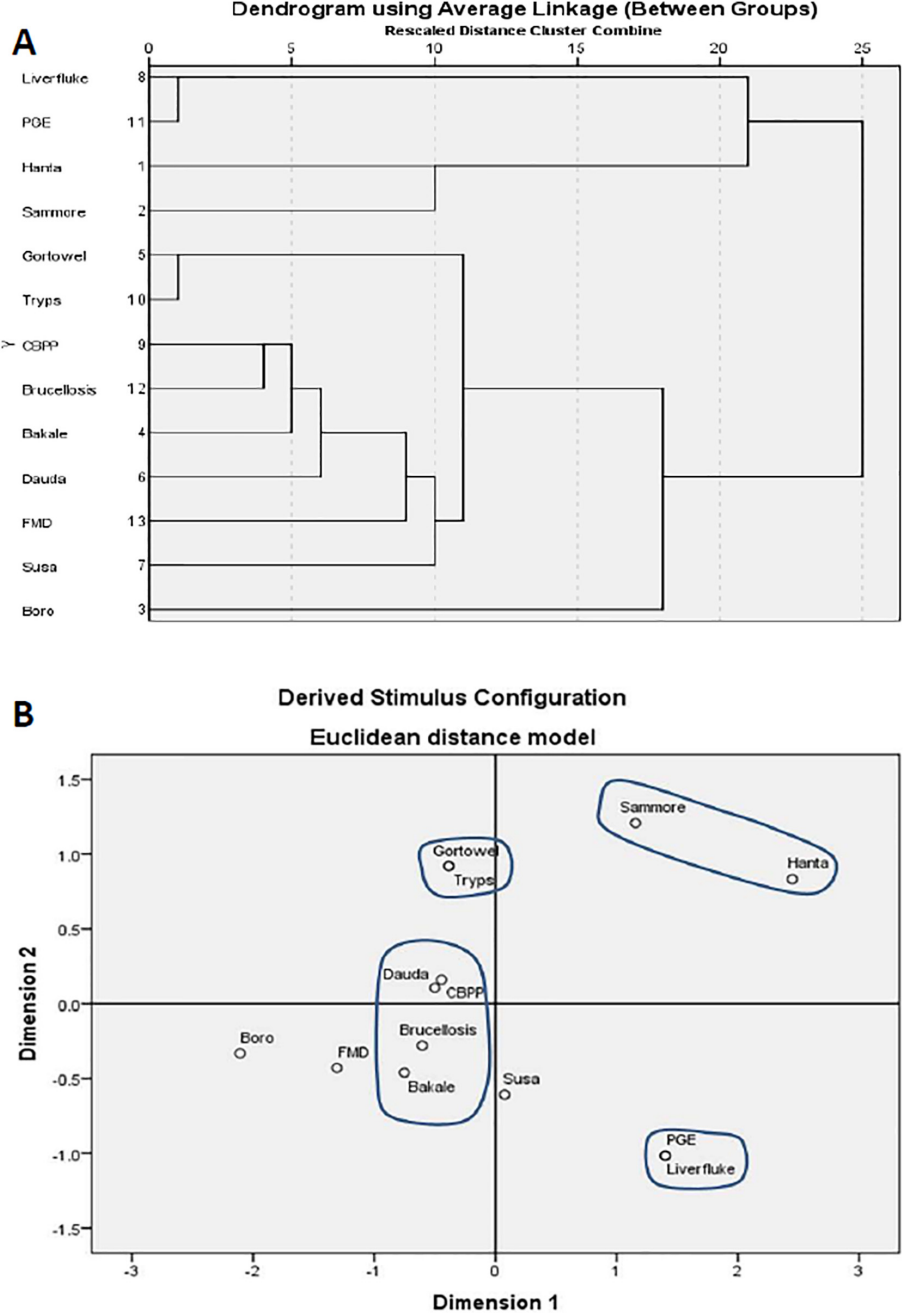
Comparison of herders' treatment profiles with standard veterinary treatments using A: hierarchical cluster analysis and B: multidimensional scaling.

When the same analysis was applied to drugs used to treat the conditions (Fig. 4), there was poor correlation between the prescribed treatments and those used by pastoralists. Prescribed treatments clustered according to causative agent/drug class (antibiotics for CBPP and brucellosis and anti-helminthics for PGE and liver fluke). There was a high correlation between treatment profiles for *Bakale* – brucellosis) and *Boro* – FMD. Susa grouped with PGE along both dimensions on MDS (albeit at low proximity). There was high proximity between treatment profiles for *Gortowel* – trypanosomiais whereas *dauda* clustered with bacterial diseases. Fulani used trypanocides to treat *gortowel*, and antibiotics for *dauda* rather than anti-helminthics prescribed for the paired diseases of liver fluke and PGE. Treatment profiles for hanta and *sammore* showed poor proximity to trypanosomiasis and CBPP but were similar to each other, with an early divergence cluster on HC and grouped along both dimensions on MDS. These treatment profiles included the widest range of drug classes recorded. Treatments for *Hanta* showed more correct choices for liver fluke than CBPP. Treatments were mostly chosen and administered by Fulani themselves. Only a third of respondents reported consulting a veterinarian.

### 3.3 Epidemiological survey

Results from the epidemiology survey showed 75.6% cattle in the survey as being infected with a tick-borne diseases (TBD), most showing mixed infections of multiple tick-borne parasites (44.3% *Anaplasma*, 62.2% *Babesia*, 37.1% *Ehrlichia*, 77.5% *Theileria*, 9.3% *Rickettsia* spp.). *Trypanosoma vivax* was the only trypanosome species identified in the survey, at 11.6% prevalence. The prevalence of PGE was 19.6% (excluding liver fluke), mostly due to *Paramphistomum spp*. (12.4%), *Oesophagostomum spp*. (6%), *Eimeria* (1.4%) and *Bunostomum* spp. (1.1%). The prevalence of liver fluke (*Fasciola hepatica)* was just 0.3%. 32% of the cattle tested had mixed infections with more than one class of parasites: 22.2% TBD and PGE; 1.8% TBD and trypanosomiasis and 8% PGE and trypanosomiasis. Table 2 shows how these compare with prevalence of diseases reported by Fulani.

**Table 2:** Prevalence of parasitic endemic diseases reported by pastoralists and discovered by bioveterinary diagnostic tests.

Disease	Reported prevalence	Disease	Test prevalence
*Hanta*	6.4%	CBPP	–
*Sammore*	8.48%	Trypanosomiasis	11.1%
*Boro*	37.9%	FMD	–
*Gortowel*	0.8%	Liver-fluke	0.3%
*Dauda*	0.4%	PGE	19.6%
*Susa*	3.8%		
Tick-borne diseases	0%	Tick-borne diseases	75%
Mixed infections	0%	Mixed infections	32%

## 4 Discussion

### 4.1 Participatory epidemiology, diagnosis and treatment

This study observed 36 distinct signs of disease recognized by the Fulani community in the study area, most of which were also used by veterinarians. Some key signs recognized by veterinarians (i.e. posture, piloerection, fever, icterus, constipation, stunting and reduced milk yield) were not considered signs of disease by the pastoralists. Reduced milk yield was considered a sign of poor nutrition but not a sign of ill-health. Others such as anaemia/pallor, sub-ventral oedema, haematuria, swollen lymph nodes were mentioned only occasionally as previously observed in other studies [18,35,39,54,55]. There was a poor distinction between dysentery and diarrhoea which are actually discrete signs associated with different diseases. Unusually, there was only a single mention of post-mortem observation of lesions or parasites. Post-mortem observations are widely used by many pastoralists to diagnose disease and their use has been previously documented amongst Fulani in Nigeria [55]. The lack of post-mortem examination in this study may be due to the common practice of selling on seriously sick animals before they die, facilitated by high market integration in the study area [41].

The prevalence of reported diseases was similar to the empirical diagnostic test results for *sammore* - trypanosomiasis and *gortowel* - liver fluke. Combined reported prevalence of *Susa* and *Dauda* were however, lower than the test results for PGE, suggestive of a high proportion of sub-clinical infections. No tick-borne diseases or mixed infections were reported by pastoralists.

This study shows good pair-wise matching of Fulani disease descriptions with classical descriptions of veterinary diseases. Hanta was the most significant disease identified by pastoralists. The standard translation for *Hanta* is liver fluke (hanta meaning “liver” in Hausa) but based on the results from this study, *Hanta* it is used to describe CBPP by Fulani pastoralists. There was moderate correlation between CBBP and *hanta* with an early divergence cluster on HC and grouping along only 1 dimension on MDS. There are two reasons for this, firstly, clinical diagnosis is more difficult for diseases with non-specific signs such *hanta* - CBPP *and sammore* - trypanosomiasis than for those with pathognomonic signs such as FMD and anthrax. Secondly, *hanta* showed two distinct syndromic profiles, those with breathing difficulty (58%) and those without (42%). Other signs were identical across both categories. CBPP shows varying clinical severity depending on the strain of the infectious agent and susceptibility of individual cattle. Many cattle in this endemic study area will have experienced previous infections, providing partial immunity for subsequent infections.

Therefore, pastoralists consider CBPP to be the most important disease in their cattle, and not liver fluke as previously reported [56,57,79]. CBPP was not included in the diagnostic survey since problems with CBPP were not previously reported in the study area. Several independent studies have indicated 27%–55% herd prevalence and 14–30% individual prevalence of CBPP amongst pastoral cattle in Nigeria [[58], [59], [60],^78^]. Pastoralists reported 72% herd prevalence and 6.4% individual prevalence of CBPP in this study. This herd prevalence was higher than previously reported [[58], [59], [60],^78^]), indicating that CBPP is a serious problem in this area. However, individual prevalence was lower as pastoralists only report active, clinical infections whereas sero-prevalence methods in published reports detect any exposure to the pathogen, whether from previous or active, clinical or subclinical infections.

CBPP is arguably the most important transboundary disease of cattle in Africa today. CBPP is an OIE listed reportable disease and a significant barrier to trade for countries where it is endemic [78]. Several national campaigns have been established to control CBPP in Nigeria, including the Joint Project (JP28) of 1980s and the 3-Year National CBPP Programme in the early 90s [61,62]. The JP28 mass vaccination programme reduced prevalence but caused severe post-vaccination reactions. Presently, control of CBPP in Nigeria focuses on containment i.e. private vaccination with T1/44 vaccine, supported by occasional state-sponsored vaccination campaigns. T1/44 confers short-term immunity and cattle must be vaccinated twice a year for 5 years to reduce prevalence. There are frequent vaccine shortages at the National Veterinary Research Institute due to quality control issues. There is also low demand for vaccines by pastoralists linked to a lack of trust after the severe post-vaccination adverse reactions during the JP28 campaign. Adverse reactions are rare with T1/44 but pastoralists remain sceptical. There is low compliance with CBPP reporting due to poor awareness of its reportable status, and poor channels for reporting.

The different meanings ascribed to hanta by pastoralists and veterinarians has important implications for surveillance. It will mask the true incidence and prevalence of CBPP cases in communities since passive surveillance reporting will record the majority of CBPP infections as liver fluke. This highlights the importance of robust participatory epidemiology methods that are validated by empirical diagnostic surveys. In the absence of robust active surveillance of endemic diseases, participatory epidemiology must be incorporated into routine passive surveillance mechanisms. This will require training and capacity building for epidemiologists and veterinary and extension staff.

There are also implications for treatment and disease control. Agro-veterinary traders, veterinarians and para-veterinarians often recommend drugs to pastoralists without physically observing the animal. Health service providers, extension officers and pharmaceutical company representatives also recommend treatment and control options for hanta based on the incorrect assumption that it is liver fluke. Pastoralists reporting hanta will be recommended anti-helminthics for liver fluke rather than vaccines or antibiotics for CBPP. The treatment profile for hanta indicates more correct options for liver-fluke (anti-helminthics) than for CBPP (antibiotics).

The study identified two previously unknown Fulfulde disease descriptors - *dauda* and *gortowel* for which pastoralists did not have Hausa equivalents. *Dauda* matches with the standard descriptions for PGE resulting in bloody diarrhoea. *Gortowel*, matched with standard descriptions for liver fluke. The prevalence of *gortowel* (0.8%) was comparable to the prevalence of liver fluke (0.3%) as detected in the fecal sampling survey.

The Fulfulde words appear to accurately describe specific disease syndromes. Fulfulde is the language of Fulani ethno-veterinary knowledge which is then translated into Hausa for everyday communication with non-Fulani (the majority of animal health providers). Fulani are likely to misapply Hausa terms or have a loose understanding of their application to animal disease. Hausa and English are the languages used for BVK and much detail can be “lost in translation”. Participatory epidemiology techniques should allow communities to express their knowledge and priorities in their own language and it is recommended that any further studies should be undertaken in Fulfulde and translated into both Hausa and English. The low agreement scores reflect the many instances of variation and “unknowns” in pastoralist responses, indicating EVK is not a single, systematic body of knowledge as confirmed by other studies [43, 37]. This highlights the importance and practical value of PE for integrating EVK and BVK to improve communication between veterinarians and pastoralists and facilitate knowledge transfer. MDS plots provide a powerful visual tool for giving community feedback and generating topical discussion.

### 4.2 Interactions between BVK and EVK

The Fulani interviewed in this study showed high levels of EVK, with good diagnostic abilities that aligned with veterinary clinical diagnosis, as reported for many pastoral groups across Africa [18,35,43]. However, their treatment strategy is based almost exclusively on veterinary drugs and not traditional EVK remedies. Veterinary drugs are preferred because they are more convenient and/or more effective than herbal preparations as found in other studies [2,36,52,63,37]. The Fulani in this study rarely use traditional remedies, except when veterinary drugs prove ineffective, also consistent with other reports [63]. However, it contrasts with contemporary Fulbe communities in Chad who mention equal use of traditional and proprietary veterinary products [43]. Treatment choices showed little association with recommended treatments and were mostly wrong, as found with other pastoralists groups [37,38,43,63,64]. Drugs from several different classes were often used to treat the same disease with little regard for product specificity. Some Fulani were not aware of the names of the drugs recommended by agro-veterinary traders. The practice of injecting animals with arbitrary mixtures of drugs was also common, particularly amongst younger pastoralists. Itinerant traders were observed on multiple occasions selling a popular proprietary mixture of ten different veterinary drugs. Despite the widespread use of veterinary drugs, only a third of respondents reported consulting a vet. The average spent on veterinary consultations per household was $15, just 0.8% of annual production costs, compared to $326 (17%) spent on drugs [65].

Fulani have low BVK, for the simple reason that this requires considerable training and experience which they do not have. Pastoralists are conscious of their own uncertainty about veterinary drugs. They freely admit that their efforts are based on trial and error and that they often depend on the recommendations of trusted agro-veterinary traders. Nevertheless, the majority persist in treating their animals themselves. This begs the question, why is this preferred this to consulting veterinarians who do have the expert knowledge required, or making concerted efforts to increase their own BVK?

The main reason for the failure to consult veterinarians is poor access – there are very few qualified private sector veterinarians practicing in Bokkos LGA and only one LGA para-veterinary officer provided by the local government for a population of 240,000 [66]. Most pastoralists have to depend on the services of private sector para-veterinarians or agro-veterinary traders. The alternative is to travel several hours to Jos or NVRI in Vom where there are several public and private sector practitioners. Even when veterinary/para-veterinary personnel are available, pastoralists view them with scepticism and therefore prefer not to consult them. Some Fulani worry that they are more interested in their fees than in the health of the livestock. Others have experienced poor treatment outcomes in the past and are not convinced of the expertise of veterinary personnel.

High EVK is in itself, a barrier to consulting vets. Studies in East Africa have shown that pastoral tribes such as the Maasai with high EVK (and larger herds in extensive systems, living further from urban centres, less westernized lifestyles and lower market integration, were less likely to consult vets and had lower BVK. In contrast, livestock owners from non-pastoral tribes such as the Koore, Arusha and Chagga with lower EVK (and smaller herds in intensive systems, living closer to urban centres, with more westernized lifestyles and higher market integration) were more likely to consult vets and had higher BVK [37,38]. The pastoral culture and high EVK of Fulani also reduce willingness to consult veterinarians. The reasons for this correlation become clear when we examine the nature of Fulani EVK. There are no specialist animal healers, instead there are varying levels of individual expertise, acquired by personal skill and experience [43]. There is prestige attached to individual EVK and it is therefore personal and protected. However, in non-pastoral groups where little prestige is attached to EVK, livestock owners are more willing to consult vets and gain more BVK in the process. They are also more willing to share knowledge with their peers [37,38].

These features of the ethnoveterinary knowledge system also contribute to unwillingness to undergo formal training for BVK. Within the Fulani community there is prestige attached to knowledge acquired by personal skill, experience that is not accorded to veterinary knowledge acquired by formal instruction or to “specialists”. Most BVK comes from individual trial and error rather than any understanding of the underlying principles of BVK. One of the biggest differences between the two systems of knowledge is that there is no common underlying theory of cause and effect for EVK as with BVK. Therefore pastoralists are less concerned with aetiology in general than with practical solutions. They have little knowledge or interest in microbe aetiology/germ theory and are not motivated to learn more [43, 38]. However, this knowledge is required for the proper use of veterinary drugs and the lack of it seriously hampers their efforts towards effective treatment of endemic disease. Fulani mostly attribute disease to factors associated with diet, environment and weather although they do have awareness of parasitic worms and insect vectors. Spiritual or mystical causes are rarely mentioned [18].

Since EVK is variable by nature, Fulani see little conflict or contradiction between the two systems and go ahead to integrate them, with poor results as seen here. They have used the “integration” approach to incorporate BVK into EVK without understanding the underlying world view, in much the same way that scientists have done with EVK in the past. Thus, there is clear evidence that the “dialogues” approach needs to be adopted by both sides for two-way knowledge transfer. There is a critical need to increase the BVK of pastoralists so that they can better manage disease in their livestock [64,67,68].

Messaging interventions have shown good results in successfully in improving BVK of rural livestock owners [64]. Within this context a messaging campaign focused on the selection and use of veterinary drugs would be very helpful, especially if integrated with improved access to local veterinarians. There is a need to develop national and international frameworks and standards for use, analysis and reporting of PE techniques [11]. Considering the reports of brucellosis, increasing adoption of PE should go hand in hand with One Health for maximum impact on health delivery and surveillance systems [[68], [69], [70]].

High profile regional and international organizations have a role to play in developing these guidelines as they can influence individual countries to develop their own tailored context-specific solutions [70,71]. Both PE and One Health would benefit from action on three fronts - challenging current, scientific expertise, reframing policy narratives and increased interaction at the research-policy-action interface [67]. In the Nigerian context, the latter is key and national organizations such as the Nigerian Veterinary Medical Association, Nigerian Medical Association and One Health Nigeria need to increase the scope and intensity of their interactions with policy makers in public health, education and capacity building.

PE facilitates two-way knowledge transfer because enhanced understanding of EVK puts vets and extension officers in a much better position to transmit BVK to livestock owners and communities in an intelligible manner. PE is also well suited to integration with conventional epidemiology as it recognizes the limits of epidemiological disease models and is a valuable tool to validate them. Indeed, mutual validation of participatory and conventional epidemiological methods is one of the central principles of PE [11,72].

Secondly, the large variation in quality of veterinary services available to pastoralists gives variable treatment outcomes for several reasons. The presence of fake and substandard drugs is one of the first reasons cited for variable treatment outcomes [4]. However, [63] have shown that improper use of these drugs by pastoralists is a bigger problem. This is also a compound issue, linked to conflicting advice on the use of veterinary drugs from veterinarians, para-veterinarians and agro-veterinary traders in consultations that take place in the absence of the sick animal. Many of these para-veterinarians and agro-veterinary traders are also not qualified to provide this advice and are likely to mislead pastoralists. Mixed infections are quite common (32%) and so several different drugs may give a positive result in the short term [18].

## 5 Conclusion

This study set out to investigate how the two knowledge systems work together for the healthcare of cattle in the study area. Results show that both systems are comparable in terms of diagnosis. As with clinical diagnosis by veterinarians, pastoralists are better able to diagnose diseases with pathognomonic signs than those with non-specific signs. Fulani mostly use veterinary drugs to treat their sick animals themselves, without consulting professionals. However, despite their high EVK, their BVK of causes and treatments of disease is quite low and most use veterinary drugs incorrectly. Given the gaps in veterinary service delivery in Nigeria, it is inevitable that pastoralists will treat their animals themselves. The most sustainable way to increase their capacity to do so correctly is two-fold. First, extension agents and veterinary service providers in both the public and private sectors must improve their ability to engage with Fulani pastoralists. The results of this study show clearly that vets need to use PE methods and published information so they can communicate effectively with pastoralists– using EVK as a starting point. The revelation of hanta as CBPP rather than liver fluke is an important contribution to this. More time needs to be spent talking about the bio-veterinary causes of disease which determine which drugs can cure specific diseases. There is a dearth of studies on Fulani beliefs about veterinary drugs, with their perceptions simply written off as “wrong”. Such studies are required for a deeper understanding of current treatment patterns, improvement in Fulani BVK and engagement with veterinary services. Secondly, there must be increased Fulani access to these improved professional veterinary services. This will provide more opportunities for dialogue and two-way knowledge transfer between vets and Fulani and will go a long way to dispense with prejudice on both sides.

## Funding

This study was jointly funded by the UK's Department for International Development (DFID) and Biotechnology and Biological Sciences Research Council (BBSRC) grant number BB/H009213/1 under the ‘Combating Infectious Diseases in Livestock for International Development’ (CIDLID) scheme.

## Authors' contributions

AOM conceived the study; AOM, CD, CI, TL planned and executed fieldwork; AOM analyzed the data; AOM and SCW prepared the manuscript. All authors read and approved the final manuscript.

## Competing interests

The authors declare that they have no competing interests.

## Ethical approval

Ethical approval for this study was obtained from the Plateau State Ministry of Agriculture. Informed consent was obtained from all participants upon enrolment into the study.
